# An elderly man with progressive focal nodular hyperplasia

**DOI:** 10.1007/s12328-019-01072-9

**Published:** 2019-11-25

**Authors:** Koichi Hamada, Satoshi Saitoh, Noriyuki Nishino, Daizo Fukushima, Kae Techigawara, Ryota Koyanagi, Yoshinori Horikawa, Yoshiki Shiwa, Hideo Sakuma, Fukuo Kondo

**Affiliations:** 1 Department of Minimally Invasive Surgical and Medical Oncology, Fukushima Medical University, 1 Hikariga-oka, Fukushima-shi, Fukushima, 960-1295 Japan; 2 Department of Gastroenterology, Southern-Tohoku General Hospital, 7-115, Yatsuyamada, Koriyama-shi, Fukushima, 963-8563 Japan; 3 Department of Hepatology, Toranomon Hospital, 2-2-2 Toranomon, Minato-ku, Tokyo, 105-8470 Japan; 4 Department of Pathology, Southern-Tohoku General Hospital, 7-115, Yatsuyamada, Koriyama-shi, Fukushima, 963-8563 Japan; 5 Department of Pathology, Teikyo University Hospital, 2-11-1 Kaga, Itabashi-Ku, Tokyo, 173-8606 Japan

**Keywords:** Focal nodular hyperplasia, Progressive type, Older age, Elderly man

## Abstract

Patients with focal nodular hyperplasia (FNH) develop benign hepatocellular nodules. FNH most frequently occurs in young women. There are no reports of the onset of FNH in elderly men. We report a case of FNH in an elderly man, whose nodules increased in number and size. The patient underwent surgery for carcinoma of the left renal pelvis at 69 years of age; no liver masses were noted on yearly follow-up contrast-enhanced computed tomography (CECT). Ten years later, CECT revealed a hepatic mass, and magnetic resonance imaging suggested FNH. The nodules increased in number and size in subsequent follow-up examinations.

## Introduction

Patients with focal nodular hyperplasia (FNH) develop benign hepatocellular nodules. FNH shows characteristic immunohistological findings without genetic mutations. On contrast-enhanced computed tomography (CECT), the nodules show a hypervascular pattern. When central scarring is present, a spoke wheel appearance is observed [1]. On ultrasound imaging, borders are unclear, and various signals are noted within the lesions. Typical contrast-enhanced ultrasound findings include a hypervascular appearance and a spoke wheel appearance [2–4]. On magnetic resonance imaging (MRI), iso-signals are often observed on T1-weighted, T2-weighted, and diffusion-weighted images. In the hepatocellular phase of gadolinium-ethoxybenzyl-diethylenetriaminpentaacetic acid-enhanced MRI (EOB-MRI), non-uniform hyperintense signals are observed and the center of the lesions are thought to reflect central scarring and vascular images [5–8].

Epidemiologically, FNH occurs more frequently in women of between 20 and 50 years of age; there have been no reports of the progression of FNH in elderly men. The natural course of FNH remains largely unclear as few reports have investigated this issue [7, 9–11]. To the best of our knowledge, this is the first reported case in which FNH occurred and the nodules increased in size and number in an elderly man. The present report describes a rare case involving the onset of FNH in an elderly man of 79 years of age, whose nodules increased in size and number at 89 years of age.

## Case report

**Fig. 1:**
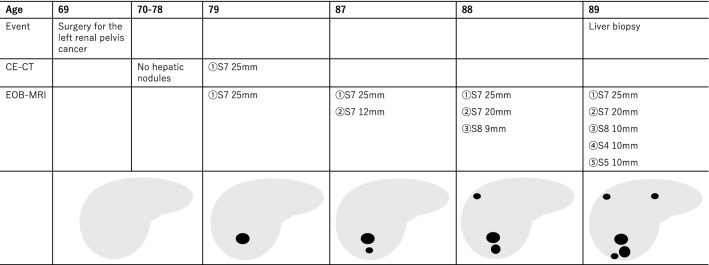
Schematic illustration of the progression of FNH. There was no hepatic nodule until he was 78 years of age. FNH appeared in segment 7 at 79 of age. The FNH nodules increased in size and number while the patient was undergoing follow-up

The patient was an 89-year-old man who had undergone curative radical resection via left nephroureterectomy for carcinoma of the left renal pelvis at 69 years of age and who was subsequently attending regular follow-up examinations. Plain and single-phase CECT scans were examined once per year. CECT, performed during a regular follow-up examination when the patient was 79 years of age, revealed a mass of 25 mm in diameter in segment 7 of the liver with uniform contrast. Ultrasound revealed a nodule with an unclear border and irregular shape and fairly hyperechoic signals. CT and ultrasound examination showed no splenomegaly. Positron emission tomography-CT (PET-CT) was performed as liver metastasis of carcinoma of the renal pelvis was suspected, but no uptake of F-18 fluorodeoxyglucose (FDG) was observed at the lesion site. As the EOB-MRI findings were suggestive of FNH, a conservative approach with regular follow-up examinations was taken. At 80 years of age, the patient developed acute myocardial infarction and his left ventricular ejection fraction (32%) was severely decreased. No changes were noted in the nodules until the patient was 87 years of age when EOB-MRI revealed a nodule of 12 mm in diameter exhibiting the same findings behind the existing FNH. The nodule was found to have increased to 20 mm in size on EOB-MRI performed when the patient was 88 years of age, and a nodule of 9 mm in diameter was also observed on the border of the right lobe. When the patient was 89 years of age, EOB-MRI again revealed 1 new nodule on the right lobe and 1 new nodule on the left lobe of the liver. Subsequently, a liver biopsy was performed (Fig. 1). We performed liver biopsy to obtain a specimen of the nodule in segment 7.

A physical examination indicated that the patient’s height, weight and body temperature was 168 cm, 58 kg, and 36.5 °C, respectively. The liver was not palpable below the costal margin, and the spleen was not palpable below the costal margin. The patient’s tumor marker and serum Mac-2-binding protein glycosylation isomer levels were within the normal ranges (Table 1). No varices were detected at endoscopy. The patient’s history of alcohol intake indicated that he was only a social drinker.

**Table 1:** Laboratory data at the time of liver biopsy

Blood cell count			Blood chemistry			Serology		
White blood cell	8000	/mm^3^	Total protein	7.0	g/dL	C-reactive protein	0.34	mg/dL
Red blood cell	463	× 10^4^/mm^3^	Albumin	3.6	g/dL	HBsAg	(–)	
Hemoglobin	13.5	g/dL	Total bilirubin	0.8	mg/dL	HCV-Ab	(–)	
Hematocrit	40.6	%	AST	20	U/L			
Platelet count	18.7	× 10^4^/mm^3^	ALT	12	U/L	Tumor markers		
			LDH	192	U/L	AFP	2.0	ng/mL
Coagulation			ALP	225	U/L	DCP	15	mAU/mL
Prothrombin time	86	%	GGTP	41	U/L	CEA	3.0	ng/mL
			Total cholesterol	147	mg/dL	CA19-9	7	U/mL
			Blood urea nitrogen	28.6	mg/dL			
			Creatinine	1.15	mg/dL	Fibrosis marker		
			Serum ammonia	60	μg/dL	M2BPGi	0.35	C.O.I

*AFP* alpha fetoprotein, *ALT* alanine aminotransferase, *AST* aspartate aminotransferase, *CA19-9* Carbohydrate antigen 19-9, *CEA* Carcinoembryonic antigen, *DCP* des-gamma carboxyprothrombin, *GGTP* gamma-glutamyl transpeptidase, *HBsAg* hepatitis B surface antigen, *HCV-Ab* hepatitis C antibody, *LDH* lactate dehydrogenase, *M2BPGi* Mac‐2 binding protein glycosylation isomer

### Computed tomography

**Fig. 2:**
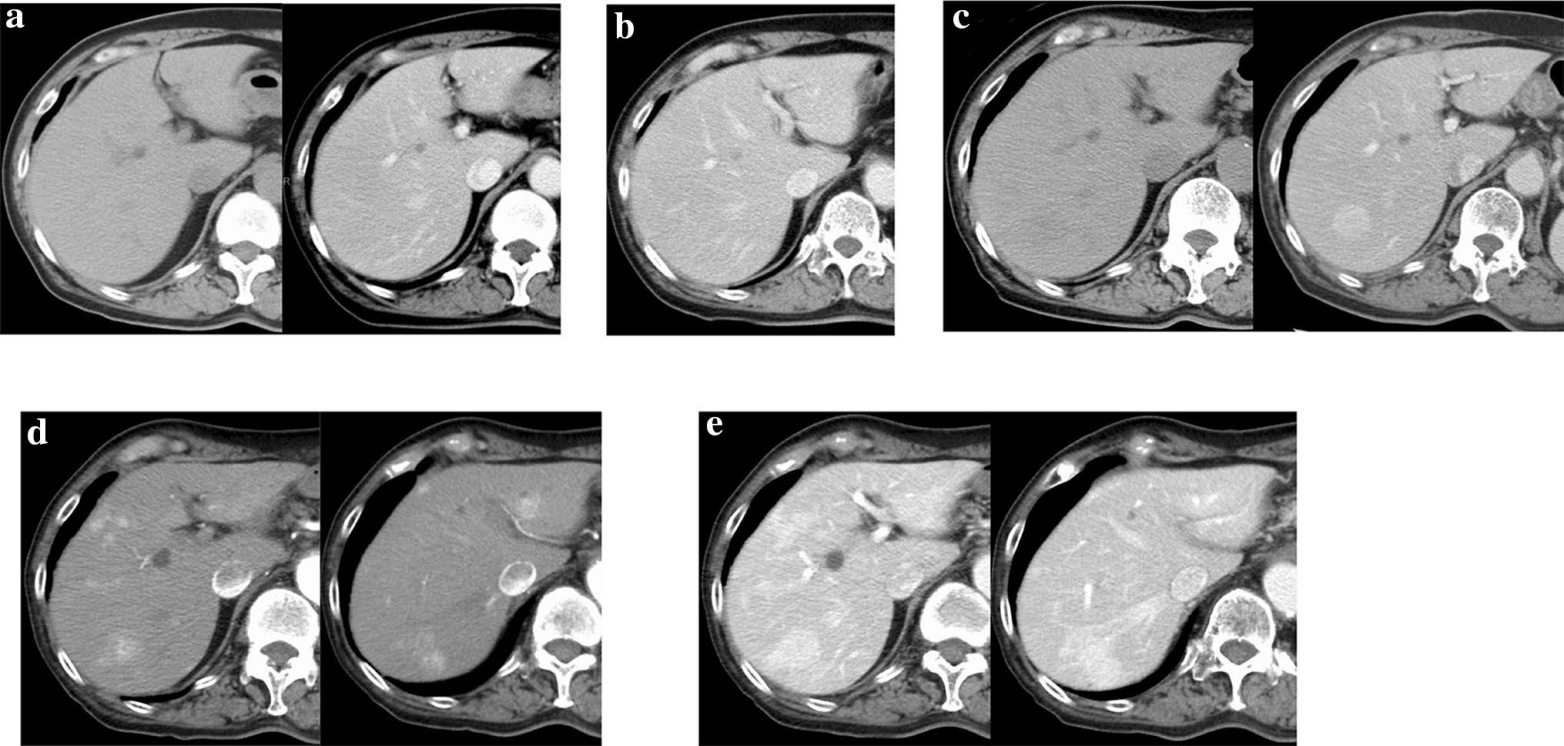
Contrast-enhanced computed tomography. **a** Plain CT and CECT at 73 years of age showed no mass lesions in the liver. **b** CECT at 77 years of age shows no mass lesions in the liver. **c** A 25-mm nodule with hyperdense and clear borders was noted in segment 7 on CECT at 78 years of age. No nodules were detected on plain CT. **d** The nodules were hyperdense in the arterial phase on CECT at 89 years of age. **e** The nodules were hyperdense in the portal phase on CECT at 89 years of age. *CECT* contrast-enhanced computed tomography

The oldest plain CT and CECT images on record, taken when the patient was 73 years of age, indicated no nodules on the liver (Fig. 2a). Liver nodules were not noted on subsequent follow-up observations after left nephroureterectomy for carcinoma of the left renal pelvis, and no hepatic nodules were observed on CECT performed when the patient was 77 years of age (Fig. 2b). A hepatic mass was first identified by CECT when the patient was 79 years of age. No hepatic mass was observed on a plain CT scan obtained at that time (Fig. 2c). CECT, performed when the patient was 89 years of age, revealed substantial enhancement of each nodule in the arterial phase (Fig. 2d) and decreased contrast effect in the equilibrium phase (Fig. 2e).

### MRI

**Fig. 3:**
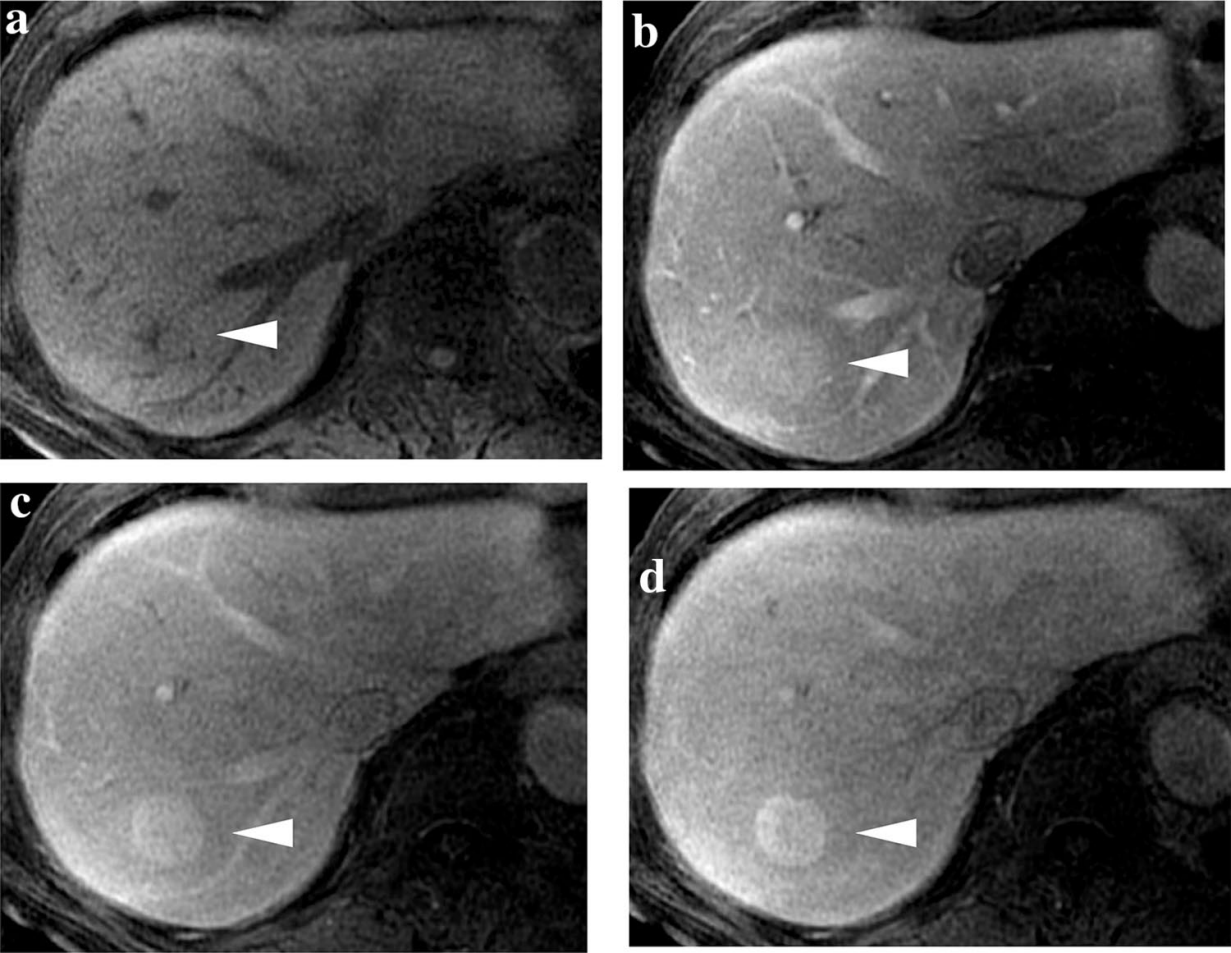
Images of multiple phase dynamic MRI on T1-weighted images for the nodule in segment 7 at 78 years of age. **a** On T1-weighted images, the lesion was hypointense (arrowhead). **b**–**d** The lesion was enhanced on dynamic MRI at 80, 120, and 180 s after contrast injection (arrowhead). *MRI* magnetic resonance imaging

We performed EOB-MRI using 1.5-T and 3.0-T imagers (GE Healthcare, Milwaukee, WI, USA). The nodule diameter on EOB-MRI, when the hepatic mass was first identified, was 25 mm. On T1-weighted images, it was hypointense, and on T2-weighted images, it had a hyperintense center. A dynamic study utilizing T1-weighted images revealed the lesion to be hypervascular (Fig. 3).

**Fig. 4:**
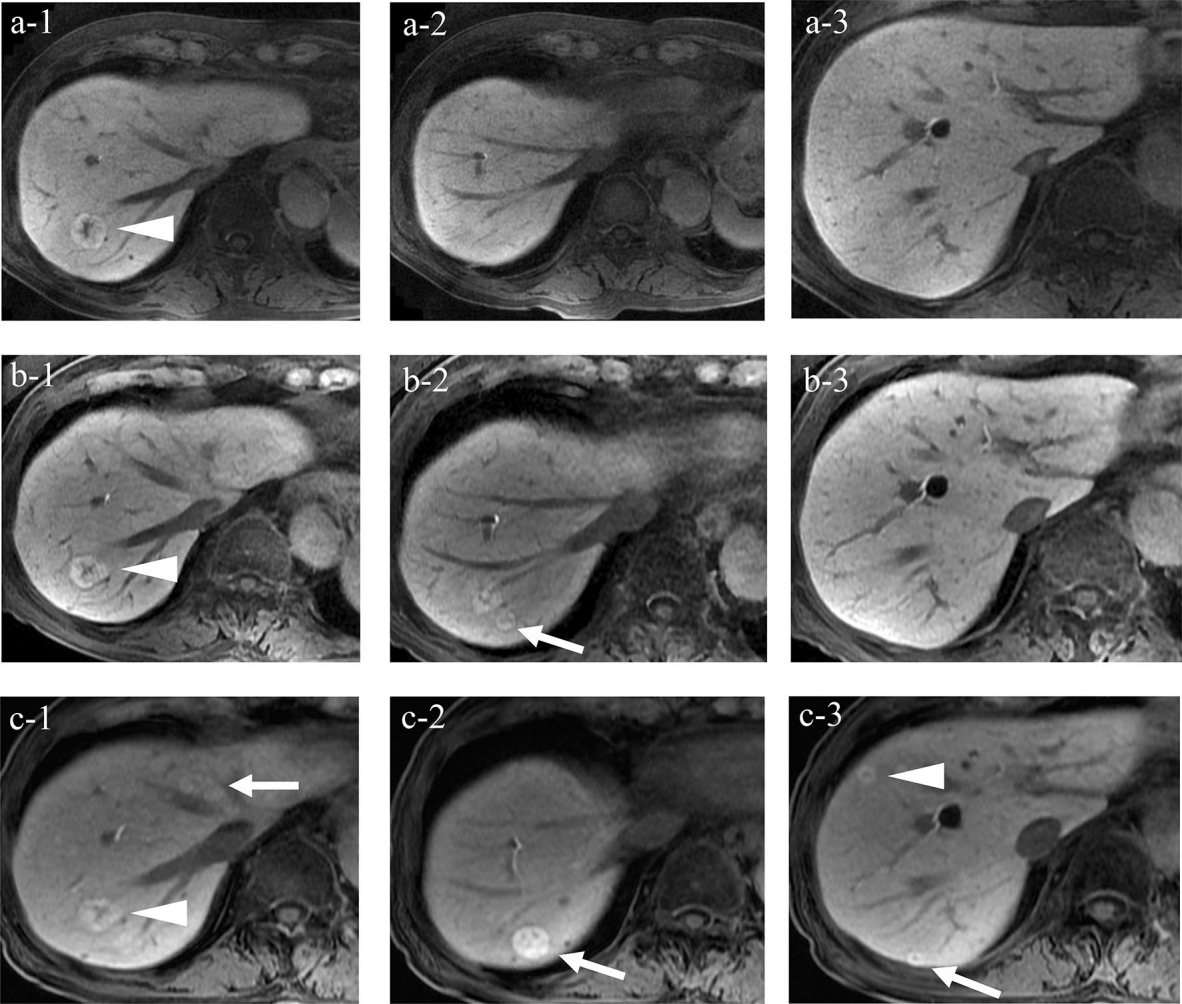
The changes in the EOB-MRI hepatobiliary phase findings over time. **a-1** At 79 years of age: a 25 mm nodule with clear borders was noted in segment 7 region with a hypointense center and a hyperintense surrounding area (arrowhead). **a-2** No mass was noted behind the nodule in segment 7. **a-3** No mass was noted in segments 7 or 8. **b-1** At 87 years of age: the nodule in segment 7 showed no major change (arrowhead). **b-2** A 12-mm nodule exhibiting similar findings was noted behind the nodule in segment 7 (arrow). **b-3** No mass was noted in segments 7 and 8. **c-1** At 89 years of age: the nodule in segment 7 showed no major change (arrowhead) and a 10-mm nodule appeared in segment 4 (arrow). **c-2** The nodule behind the nodule in segment 7 increased to 20 mm (arrow). **c-3** Nodules of 10 mm in diameter appeared in segments 7 (arrow) and 8 (arrowhead). *EOB-MRI* gadolinium-ethoxybenzyl-diethylenetriaminpentaacetic acid-enhanced magnetic resonance imaging

In the hepatobiliary phase, the nodule center was hypointense and surrounded by hyperintense signals, indicating a central scar. The 12-mm FNH that appeared when the patient was 87 years of age had increased to 20 mm in diameter, with non-uniform hyperintense signals observed in the hepatobiliary phase when he was 88 years of age. New lesions were observed in the anterior and posterior segment borders of the right lobe. In the hepatobiliary phase, the nodule center was hypointense and surrounded by hyperintense signals. Over time, the FNHs increased in number and size (Fig. 4).

### Ultrasound (Fig. 5)

**Fig. 5:**
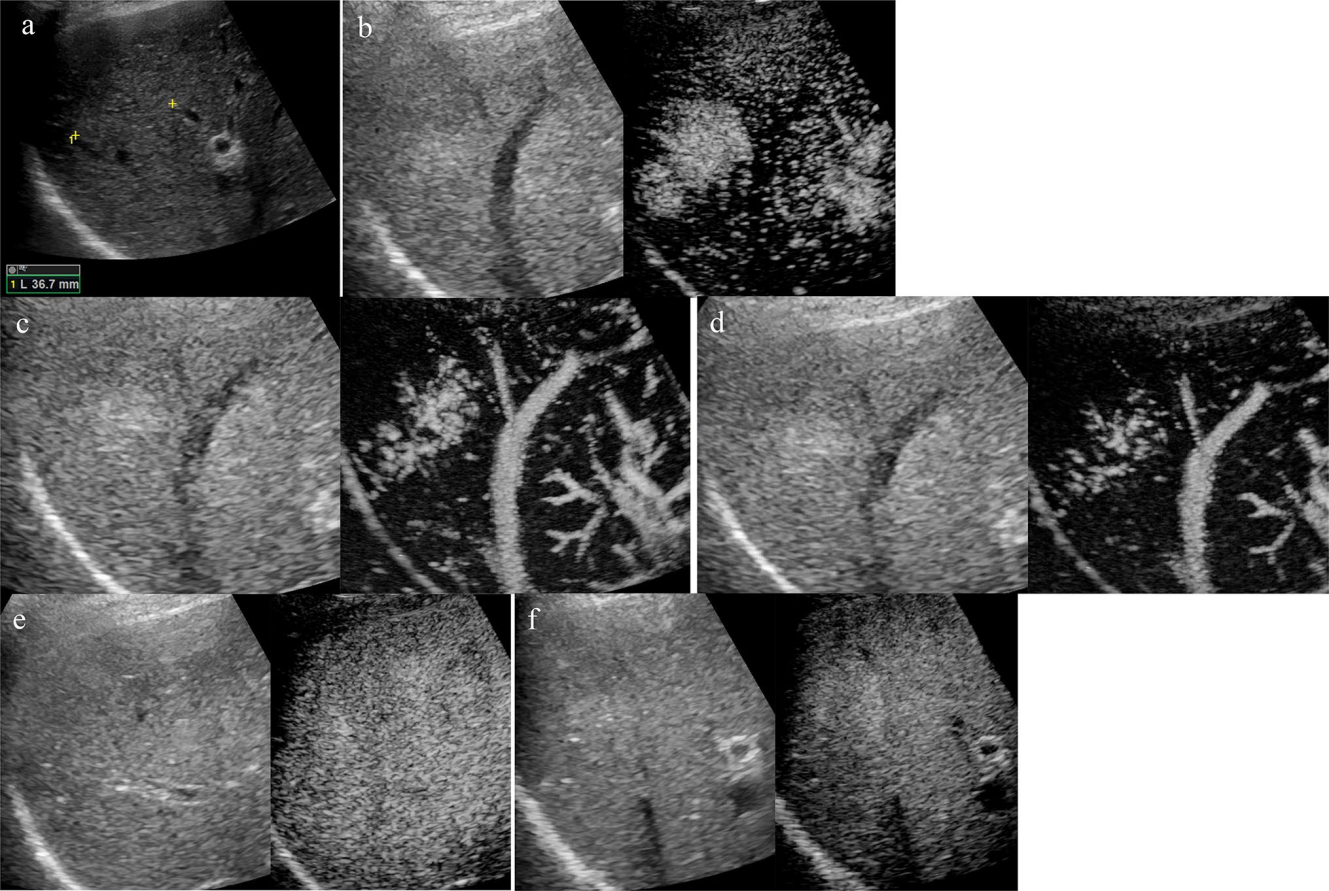
Ultrasound examination. **a** B-mode ultrasound showed a 35-mm mass lesion as a slightly hyperechoic area with unclear borders in segments 6 and 7. **b** Contrast-enhanced ultrasound showed that the entire lesion was enhanced in the arterial phase. **c, d** Perfusion images showed that the enhancement pattern of fast centrifugal filling of the lesion was composed of central vessels and radical vascular branches (spoke-wheel appearance). **e** In the post-vascular phase, there was no defect in the lesion. **f** The mass became iso-enhanced in the portal phase

An ultrasound examination (Logiq E9, GE Healthcare, Milwaukee, WI, USA), performed during the liver biopsy, revealed a nodule of 35 mm in diameter with unclear borders, an irregular shape, and fairly hyperechoic signals in segment 7 on B mode images. The nodule had no halo. We did not note any posterior echo enhancement or lateral shadows (Fig. 5a). Color Doppler and pulse wave Doppler ultrasound revealed arterial flow from the nodule center. The use of Sonazoid (Daiichi-Sankyo, Tokyo, Japan) contrast agent revealed that the nodules were hypervascular, spreading out from the nodule center on the arterial phase (Fig. 5b).

Perfusion images indicated that the enhancement pattern of fast centrifugal filling of the lesion was composed of central vessels and radical vascular branches (the so-called spoke-wheel appearance) (Fig. 5d).

In the post-vascular phase (Kupffer phase), there was no defect in the lesion (Fig. 5e).

### Histological and immunohistochemical findings (Fig. 6)

**Fig. 6:**
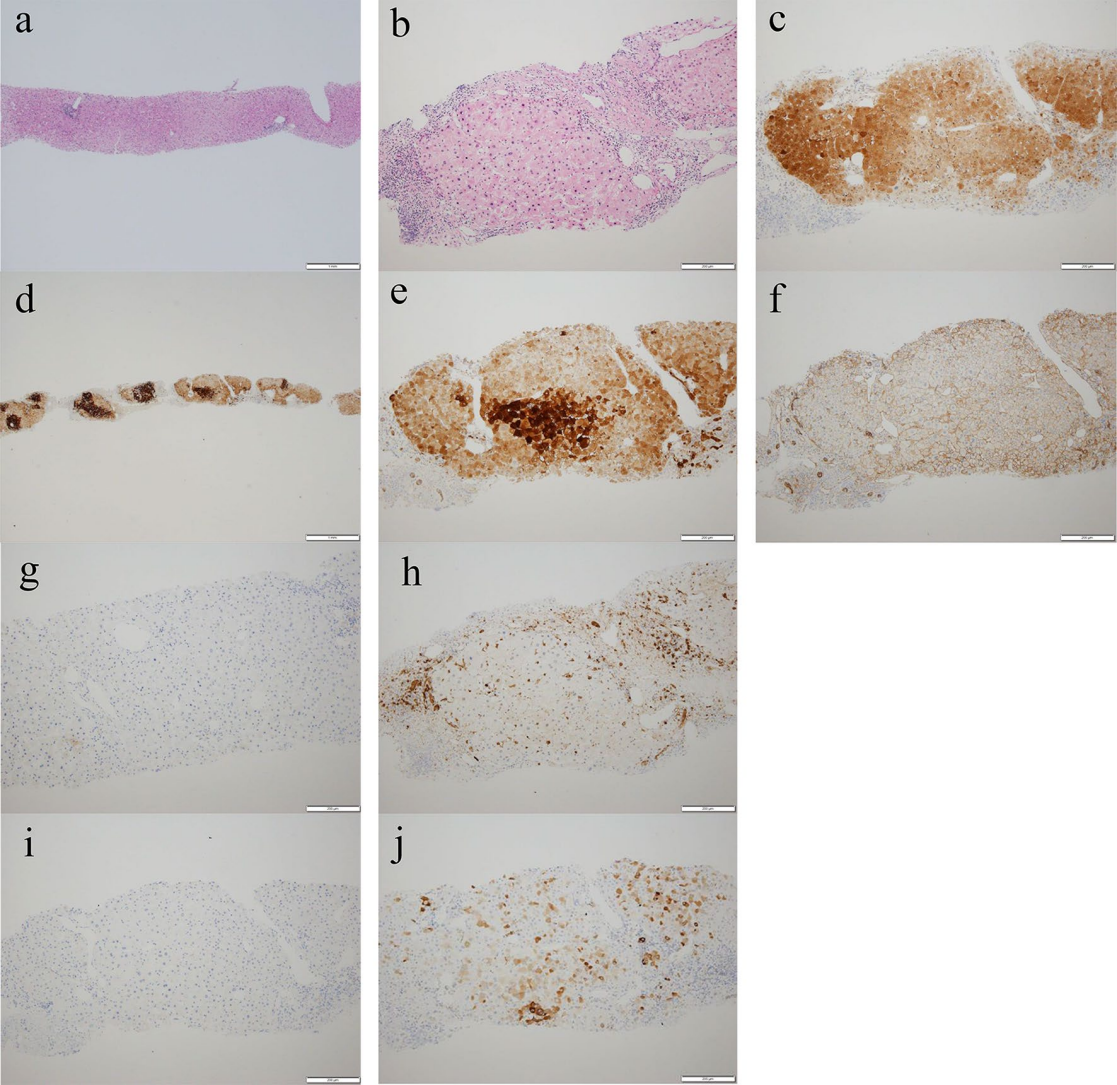
The histological and immunohistochemical findings. **a** Hematoxylin–eosin staining revealed that the background liver was normal (bar = 1 mm). **b** Hematoxylin–eosin staining revealed that the portal veins and sinusoids were partially. dilated and the infiltration of lymphocytes in the lesion (bar = 200 μm). **c** Liver-fatty acid-binding protein: positive (bar = 200 μm). **d** Immunolabelling for glutamine synthetase reveals map-like staining within the lesion (bar = 1 mm). **e** Immunolabelling for glutamine synthetase reveals map-like staining within the lesion (bar = 200 μm). **f** β-catenin was not positively stained in the nuclei of the hepatocytes (bar = 200 μm). **g** Serum amyloid A: negative (bar = 200 μm). **h** Heat shock protein 70: negative (bar = 200 μm). **i** Glypican 3: negative (bar = 200 μm). **j** Staining of C-reactive protein was not significantly positive (bar = 200 μm)

The background liver was normal (Fig. 6a). The histological and immunohistochemical findings were compatible with FNH. Staining for liver-fatty acid-binding protein (L-FABP) was positive (Fig. 6c). Immunolabelling for glutamine synthetase revealed map-like staining within the lesion (Fig. 6d, e). Staining for β-catenin (Fig. 6f), serum amyloid A (SAA) (Fig. 6g), heat shock protein 70 (HSP70) (Fig. 6h), and Glypican3 (GPC3) (Fig. 6i) was negative. Staining of C-reactive protein (CRP) was not significantly positive. (Fig. 6j).

Histological findings showed that there was no recurrence of carcinoma of the renal pelvis.

## Discussion

FNH is a hyperplastic lesion with a hypervascular appearance on various images. Previously, it had been difficult to differentiate FNHs from hypervascular hepatocellular carcinomas; thus, hepatectomy was commonly performed. In addition, it tends to be difficult to differentiate FNHs from hepatocellular adenomas (HCA). However, advances in diagnostic imaging and pathology have made it possible to definitively diagnose the condition with biopsy, and a conservative approach with regular follow-up examinations is now commonly selected. According to the WHO Classification of Tumors of the Digestive System 2010, HCA can be classified by genetic type into 4 subtypes [12]. HCA can be clearly defined by means of mutated genes, immunohistochemical findings, gender-related differences, histological characteristics, and typical clinical findings. While no genetic mutations are noted in cases of FNH, immunohistochemistry typically reveals that GS has a map-like pattern, and the lesions are L-FABP-positive, SAA-negative, and β-catenin-negative. It is also typical for histological examinations to reveal a central scar, for the normal portal region to not be apparent, and for inflammatory cell invasion, ductular reactions, and sinusoid expansion to be noted [13].

Our patient developed FNH while undergoing follow-up after surgery for carcinoma of the renal pelvis. There was no hepatic mass on CECT at 78 years of age. The following year, CECT showed a 25-mm hypervascular mass in the right lobe of the liver. In this extremely rare case, we observed increases in the size and number of FNHs while following the patient for FNH. The patient’s diagnostic imaging findings were suggestive of FNH and the condition was diagnosed pathologically. Background factors related to the development of benign hepatocellular nodules include FNH, nodular regenerative hyperplasia, and partial nodular transformation. These lesions are caused by anomalous components of the portal tract and are called “anomalous portal tract syndrome” [14, 15]. FNH is an arterial dominant nodule caused by Glisson’s capsule abnormalities.

Multiple FNHs commonly complicate cases of congenital heart disease or liver disease with blood flow abnormalities. It has been reported that FNH may arise in cases of congenital heart disease following Fontan surgery for cases of the single cardiac ventricle [16]. Liver diseases accompanied by blood flow abnormalities include non-cirrhotic portal hypertension, such as Budd–Chiari syndrome, idiopathic portal hypertension, nodular regenerative hyperplasia, and extrahepatic obliteration [14, 15, 17, 18].

A Glisson’s capsule abnormality might have played a role in the development of FNH. However, our case did not involve congenital heart disease or liver disease with blood flow abnormalities; thus, the cause of the increase in the size and number of lesions was unknown.

While most aspects of the natural course of FNH remain unknown, a small number of cases have been reported. Two reports said that FNHs increased in 2 of 18 cases [19] and 1 of 34 cases [20]. We found that each report indicated that it was rare for the FNHs to increase in size. The condition in which FNH nodules increase in size is referred to as progressive-type FNH and is a rare clinical finding. One possible reason for FNH nodules to increase in size is an increased amount of blood flow into the lesion [21, 22]. Several reports targeting female patients have indicated correlations with the use of oral contraceptives (OCs) and pregnancy, with 1 report suggesting that the long-term use of OCs is related to an increase in the size of FNH nodules [23]. It has also been reported that temporarily stopping OCs resulted in the regression of a giant FNH nodule [24]. It has been reported that FNH nodules may complicate cases of hepatocellular adenoma [17]. The Armed Forces Institute of Pathology reported the malignant transformation of FNH [25, 26]. These reports suggested that there was a possibility that FNH may transform into other types of nodules. In this case, the only pathological evidence was from a biopsy specimen. Thus, we cannot deny the possibility that HCA or malignancy existed with FNH in another area. Further follow-up investigations should be conducted.

FNH occurs more frequently in women of between 20 and 50 years of age. The abovementioned reports included a large proportion of female patients of 15–54 years of age [19] and 16–63 years of age [20], respectively, with no elderly cases, such as our patient.

While the cause of progression of FNH in our patient remains unclear, we reported a very rare case in which FNH nodules increased in size and number in an elderly man.

We reported a valuable case involving an elderly male patient with FNH who was observed from a time in which liver mass was observed on imaging. In the present case, FNH appeared and the size and number of the nodules increased during the observation period.
